# The mechanism of loop C-neonicotinoid interactions at insect nicotinic acetylcholine receptor **α**1 subunit predicts resistance emergence in pests

**DOI:** 10.1038/s41598-020-64258-z

**Published:** 2020-05-05

**Authors:** Shota Shimada, Masaki Kamiya, Sho Shigetou, Kakeru Tomiyama, Yuma Komori, Leo Magara, Makoto Ihara, Kazuhiko Matsuda

**Affiliations:** 1 Department of Applied Biological Chemistry, Faculty of Agriculture, Kindai University, 3327-204 Nakamachi, Nara, 631-8505 Japan; 2 Agricultural Technology and Innovation Research Institute, Kindai University, 3327-204 Nakamachi, Nara, 631-8505 Japan

**Keywords:** Chemical biology, Neuroscience, Environmental sciences

## Abstract

Neonicotinoids selectively modulate insect nicotinic acetylcholine receptors (insect nAChRs). Studies have shown that serine with ability to form a hydrogen bond in loop C of some insect nAChR α subunits and glutamate with a negative charge at the corresponding position in vertebrate nAChRs may contribute to enhancing and reducing the neonicotinoid actions, respectively. However, there is no clear evidence what loop C properties underpin the target site actions of neonicotinoids. Thus, we have investigated the effects of S221A and S221Q mutations in loop C of the *Drosophila melanogaster* Dα1 subunit on the agonist activity of imidacloprid and thiacloprid for Dα1/chicken β2 nAChRs expressed in *Xenopus laevis* oocytes. The S221A mutation hardly affected either the affinity or efficacy for ACh and imidacloprid, whereas it only slightly reduced the efficacy for thiacloprid on the nAChRs with a higher composition ratio of β2 to Dα1 subunits. The S221Q mutation markedly reduced the efficacy of the neonicotinoids for the nAChRs with a higher composition of the β2 subunit lacking basic residues critical for binding neonicotinoids. Hence, we predict the possibility of enhanced neonicotinoid resistance in pest insect species by a mutation of the serine when it occurs in the R81T resistant populations lacking the basic residue in loop D of the β1 subunit.

## Introduction

Neonicotinoids are insecticides that modulate competitively insect nAChRs^1–3^. They represent high selective toxicity to insect over vertebrate nAChRs and show high selective toxicity to insects with diverse actions^1–6^. They show high systemicity in crop plants, enabling seed treatments and now make up >25% of global pesticide sales^7^. Their potential risks to non-target pests, including pollinators, has been demonstrated^8^. The use of some neonicotinoids in the field is now restricted in the EU^9,10^. It remains of interest to understand the mechanism of target-site actions of neonicotinoids in order to assist in the design of new, more eco-friendly pesticides.

Studies of target site actions show that basic residues in loop D of the nAChRs binding site play a key role in electronic interactions with the nitro or cyano group of neonicotinoids^1,2,5,11–13^. Indeed, the R81T mutation in loop D of aphids was first predicted^13^ then shown^14^ to reduce the affinity of neonicotinoids, thus resulting in resistance. We found earlier that a mutation of serine at position 221 to glutamate in the YXCC motif in loop C of the fruitfly (*D. melanogaster*) Dα1 subunit also markedly reduced the agonist action of imidacloprid and thiacloprid on Dα1/chicken β2 hybrid nAChR expressed in *Xenopus laevis* oocytes, pointing to a contribution of the serine221 to the selective action of the neonicotinoids tested^15^. A plausible explanation of this result is that repulsion of the electrostatically negative nitro group of imidacloprid and cyano group of thiacloprid by electronegative glutamate residue led to the reduced agonist actions. However, this mutation also changes the size of the residue.

**Figure 1:**
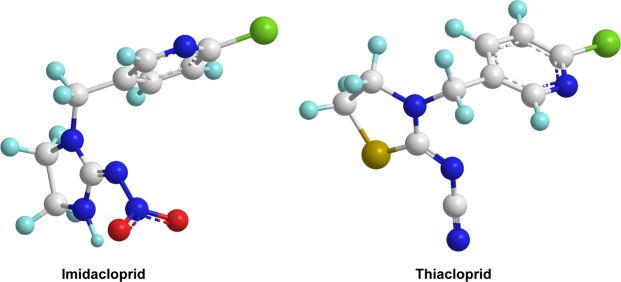
Imidacloprid and thiacloprid tested in this study, Figures are illustrated as ball and sticks where carbons, hydrogens, nitrogens, chlorine and sulfur are colored white, sky blue, blue, light green and yellow, respectively. Delocalized double bonds are shown as broken lines. Each chemical structure was drawn using Chem 3D combined with Chem Draw (PerkinElmer, Waltham, MA, USA).

In this study, the Ser221 of the Dα1 subunit was mutated to alanine or glutamine and then imidacloprid and thiacloprid as representatives of neonicotinoids carrying a nitro and cyano group, respectively (Fig. 1), were tested on the wild type and mutant Dα1β2 AChRs with two different subunit composition ratios to clarify the role for Ser221 in the selective interactions with neonicotinoids.

## Methods

### Mutations of cDNA and preparation of cRNAs

All experimental protocols for preparing recombinant DNAs were approved by Kindai University. cDNAs of the fruitfly Dα1 (Accession number NM_079757) and chicken β2 subunit (Accession number NM_204813) were cloned into the pcDNA3.1 (+) vector (Thermo Fisher Scientific, Waltham, MA, USA). The S221A mutation was introduced with forward primer 5′- TTCTACGCATGCTGCGAGGAGCCG-3′, reverse primer 5′- GCAGCATGCGTAGAACTTCTCGTT-3′, whereas the S221Q mutations was introduced with forward primer 5′- TGCTGCGAGGAGCCGTATCTGGACA-3′, reverse primer 5′- CTGGTAGAACTTCTCGTTCCGCACC-3′. The entire nucleotide sequence of the S221A mutant cDNA was confirmed by automated sequencing using a 3130xl genetic analyzer (Thermo Fisher Scientific).

Each cDNA construct was linearized by digesting with *Xho*I. cRNAs encoding wild type and mutant Dα1 subunits as well as chicken β2 subunit was prepared from the cDNA construct using a mMESSAGE mMACHINE T7 ULTRA Kit (Thermo Fischer Scientific). cRNA was dissolved in RNase free water at a concentration of 1 μg/μL. To express (Dα1)_3_(β2)_2_ and (Dα1)_2_(β2)_3_ nAChRs in *Xenopus* oocytes, cRNAs of Dα1 and β2 subunits were mixed at ratios of 5:1 and 1:5, respectively^15,16^.

Female frogs (*X. laevis*) were anesthetised and oocytes were obtained according to the U.K. Animals (Scientific Procedures) Act, 1986. After treatment with collagenase and the follicle cell layers were removed from oocytes^6,13,17,18^. Each oocyte was injected with 50 nL of the cRNA solution and incubated for 4 or 5 days at 16 °C in the standard oocyte saline (SOS, pH 7.6) supplemented with penicillin (100 units/mL), streptomycin (100 μg/mL), gentamycin (20 μg/mL) and sodium pyruvate (2.5 mM) at 16 °C^15,16^. Each data was obtained using oocytes from at least two frogs.

### Voltage-clamp electrophysiology

Agonist activity of ACh and neonicotinoids were evaluated by voltage-clamp electrophysiology as previously described. Oocytes were secured in a chamber and perfused with a SOS containing 0.5 μM atropine at a flow rate of 5–10 mL/min^6,13,17^. Two glass electrodes containing 2 M KCl were impaled into oocytes and oocyte membrane currents in response to bath applied agonists were recorded using an Axoclamp 900 A amplifier with Clampex 10 (Molecular Devices, San Jose, CA, USA) at a membrane potential of −100 mV. The membrane current data were digitized by a Digidata 1550B A/D converter (Molecular Devices) and analyzed by Clampfit 10 (Molecular Devices).

The compound was bath-applied in SOS for 3–5 s at an interval of 3–5 min with increasing agonist concentrations^15,16^. When recording the peak amplitude of the responses to imidacloprid and thiacloprid at concentrations higher than 1 μM, one oocyte was used for recording one response and abandoned to prevent underestimation of agonist responses resulting from irreversible nAChR desensitization.

### Data fitting

Peak current amplitude of each response to all the agonists were normalized to that of the response to 100 μM ACh and fitted by non-linear regression using a Prism 6 (GraphPad software, San Diego, CA, USA) according to the following equation:

$${\rm{Y}}=\frac{{{\rm{I}}}_{{\rm{\max }}}}{{1+10}^{({{\rm{\log }}{\rm{EC}}}_{50}-{\rm{X}}){n}_{{\rm{H}}}}}$$

where I_max_ is the maximum normalized response, EC_50_ is the half maximal response, X is the log [agonist concentration (M)] and n_H_ is the Hill coefficient. Experiments were repeated (n = 5). I_max_ and pEC_50_ (= −logEC_50_) values were obtained as mean ± standard error of the mean. Concentration-response curve illustration and statistical analyses were performed using Prism 6.

### Homology modeling

The homology models of wild type and S221A mutant of the orthosteric agonist binding domain of (Dα1)_3_(β2)_2_ nAChR complexed with thiacloprid were built using Modeller^19^ with the structure coordinate of acetylcholine binding protein from *Lymnaea stagnalis* complexed with thiacloprid (PDB id 3WTK)^20^. Amino acid sequences of Dα1 and β2 subunits were aligned with the sequence of AChBP, and the models in complex with thiacloprid were built using automodel algorism of Modeller by taking into considerations water molecules observed frequently in the AChBP-neonicotinoid complexes. Models are illustrated using PyMOL 2.3 (Schrödinger, New York, NY, USA).

## Results and Discussion

### Effects of S221A and S221Q mutations on the agonist activity of ACh

**Figure 2:**
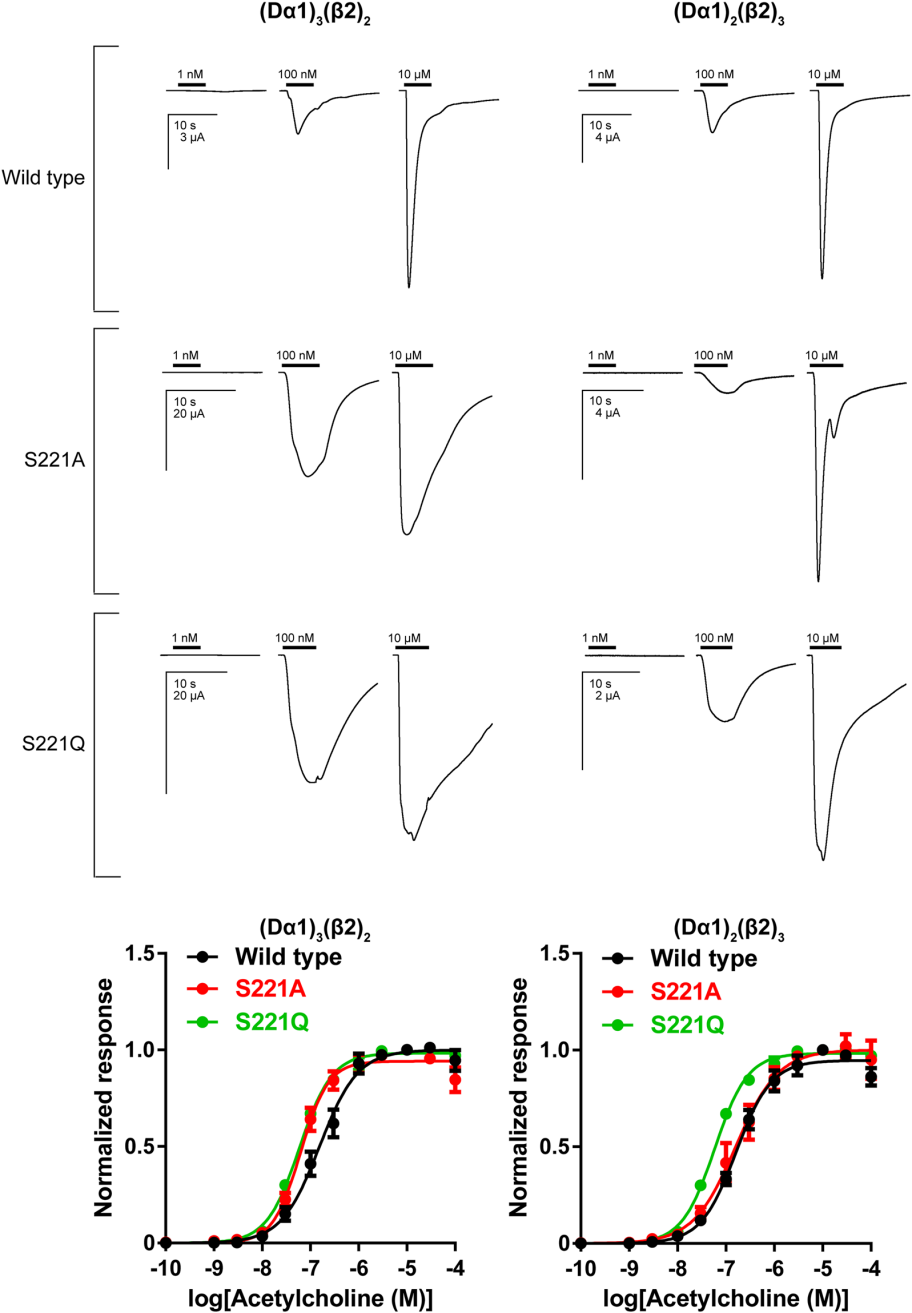
Agonist actions of ACh on wild type, S221A mutant and S221Q mutant of (Dα1)_3_(β2)_2_ and (Dα1)_2_(β2)_3_ nAChRs expressed in *X. laevis* oocytes. Current responses to ACh for the nAChRs tested are shown above concentration-response data. Horizontal lines indicate bath applications of ACh. Each data plotted represents mean ± standard error of the mean (n = 5).

First, we investigated the agonist action of ACh on the wild type, S221A mutant and S221Q mutant Dα1β2 nAChRs. ACh activated wild type (Dα1)_3_(β2)_2_ and (Dα1)_2_(β2)_3_ nAChRs concentration-dependently with pEC_50_ values of 6.81 ± 0.05 and 6.79 ± 0.04 (Fig. 2, Table 1). The S221A and S221Q had a minimal effect on the pEC_50_ value for the (Dα1)_3_(β2)_2_ and (Dα1)_2_(β2)_3_ nAChRs (Fig. 2, Table 1). Hence, the effects of these mutations on the neonicotinoid actions, if any, can be interpreted as the result of changes in the selective interactions with neonicotinoids.

**Table 1:** Agonist action of ACh on the (Dα1)_3_(β2)_2_ and (Dα1)_2_(β2)_3_ nAChRs expressed in *X. laevis* oocytes^a^. ^a^Data are represented as mean ± standard error of the mean (n = 5).

	(Dα1)_3_(β2)_2_		(Dα1)_2_(β2)_3_	
	pEC_50_	I_max_	pEC_50_	I_max_
Wild type	6.81 ± 0.05	0.999 ± 0.020	6.79 ± 0.04	0.947 ± 0.018
S221A	7.20 ± 0.04	0.942 ± 0.016	6.80 ± 0.07	1.001 ± 0.031
S221Q	7.23 ± 0.02	0.984 ± 0.008	6.57 ± 0.05	0.961 ± 0.021

### Effects of S221A and S221Q mutations on the agonist activity of neonicotinoids

**Figure 3:**
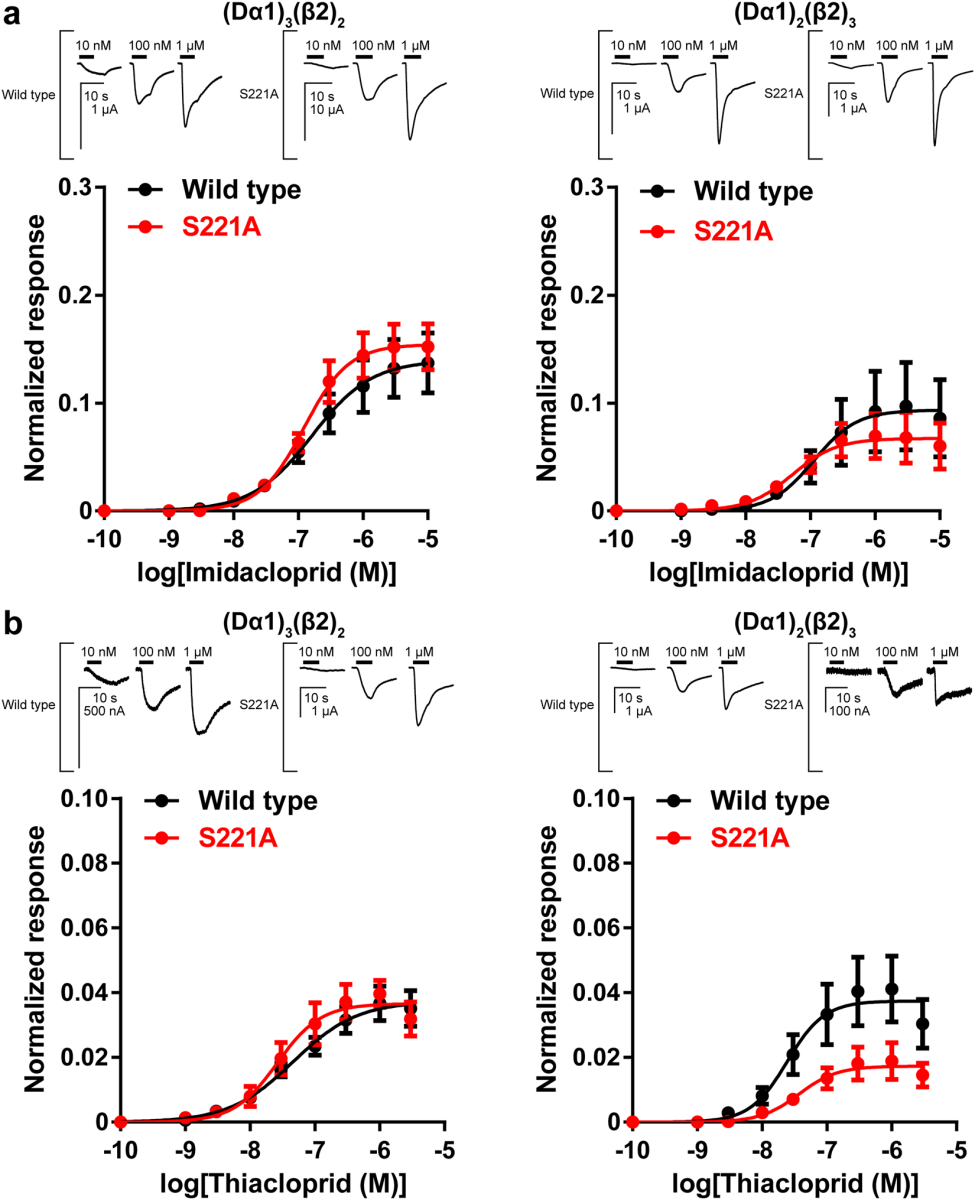
Agonist actions of imidacloprid and thiacloprid on wild type and S221A mutant Dα1β2 nAChRs expressed in *X. laevis* oocytes. In (A) and (B), inward current oocytes expressing the nAChRs in response to imidacloprid are shown above the concentration-response curves. Horizontal lines indicate bath applications of imidacloprid and thiacloprid. (**a**) Concentration-normalized response relationships of imidacloprid for the wild type and mutant (Dα1)_3_(β2)_2_ and (Dα1)_2_(β2)_3_ nAChRs. (**b**) Concentration-normalized response relationships of thiacloprid for the wild type and mutant (Dα1)_3_(β2)_2_ and (Dα1)_2_(β2)_3_ nAChRs. Each data plotted represents mean ± standard error of the mean (n = 5).

Given the evidence that the S221A mutation in the Dα1 subunit had only a minor impact on the agonist action of ACh on the Dα1β2 nAChRs, we tested imidacloprid and thiacloprid on the wild type and S221A mutant nAChRs. Imidacloprid increased inward currents concentration-dependently in oocytes expressing the (Dα1)_3_(β2)_2_ and (Dα1)_2_(β2)_3_ nAChRs (Fig. 3a). Thus, the peak amplitude was normalized by the amplitude of the 10 μM ACh response and the concentration-response data were fitted to obtain the pEC_50_ and Imax values. In contrast with the effects of the S221E mutation that markedly reduced the affinity in terms of the pEC_50_ value of imidacloprid^15^, the S221A mutation hardly affected the agonist activity of the compound, irrespective of the subunit composition ratio (Fig. 3a, Table 2).

**Table 2:** Agonist actions of imidacloprid and thiacloprid on the (Dα1)_3_(β2)_2_ and (Dα1)_2_(β2)_3_ nAChRs expressed in *X. laevis* oocytes^a^.

	Imidacloprid				Thiacloprid			
	(Dα1)_3_(β2)_2_		(Dα1)_2_(β2)_3_		(Dα1)_3_(β2)_2_		(Dα1)_2_(β2)_3_	
	pEC_50_	I_max_	pEC_50_	I_max_	pEC_50_	I_max_	pEC_50_	I_max_
Wild type	6.79 ± 0.15	0.140 ± 0.015	6.95 ± 0.28	0.094 ± 0.015	7.34 ± 0.15	0.037 ± 0.003	7.61 ± 0.18	0.037 ± 0.004
S221A	6.91 ± 0.10	0.154 ± 0.009	7.28 ± 0.22	0.067 ± 0.008	7.58 ± 0.13	0.037 ± 0.003	7.43 ± 0.19	0.017 ± 0.002
S221Q	7.10 ± 0.22	0.073 ± 0.009	7.28 ± 0.08	0.022 ± 0.001*^d^	7.51 ± 0.17	0.023 ± 0.002*	7.56 ± 0.14	0.0060 ± 0.0005*
S221E^b^	ND^c^	ND	6.46 ± 0.24	0.116 ± 0.014	6.16 ± 0.22	0.085 ± 0.012	6.94 ± 0.24	0.035 ± 0.005

^a^Data are represented as mean ± standard error of the mean (n = 5, >2 frogs).

^b^Cited from reference^14^.

^c^ND, could not be determined because the concentration response curve did not reach a plateau.

^d^Asterisk (*) indicates that the I_max_ value for the mutant nAChR is significantly different from that for the wild type nAChR (one-way ANOVA, Bonferroni-test, *P* < 0.05).

Thiacloprid evoked lower amplitude response than imidacloprid in oocytes expressing the nAChRs tested. The S221A mutation only slightly reduced the I_max_ value of thiacloprid for the nAChRs with increased β2 subunit composition ratio (Fig. 3b, Table 2, *P* > 0.05 by one-way ANOVA (Bonferroni-test), but *P* < 0.05 by one-tailed t-test). The result suggests that thiacloprid does not rely mainly on the hydrogen bond with Ser221 when it binds to the Dα1/Dα1 subunit interface containing basic residues in loop E and loop G^1,15,16^, but does when it binds to the Dα1/β2 subunit interface lacking basic residues necessary for the interactions with neonicotinoids^1,2,5,11,13,20^.

**Figure 4:**
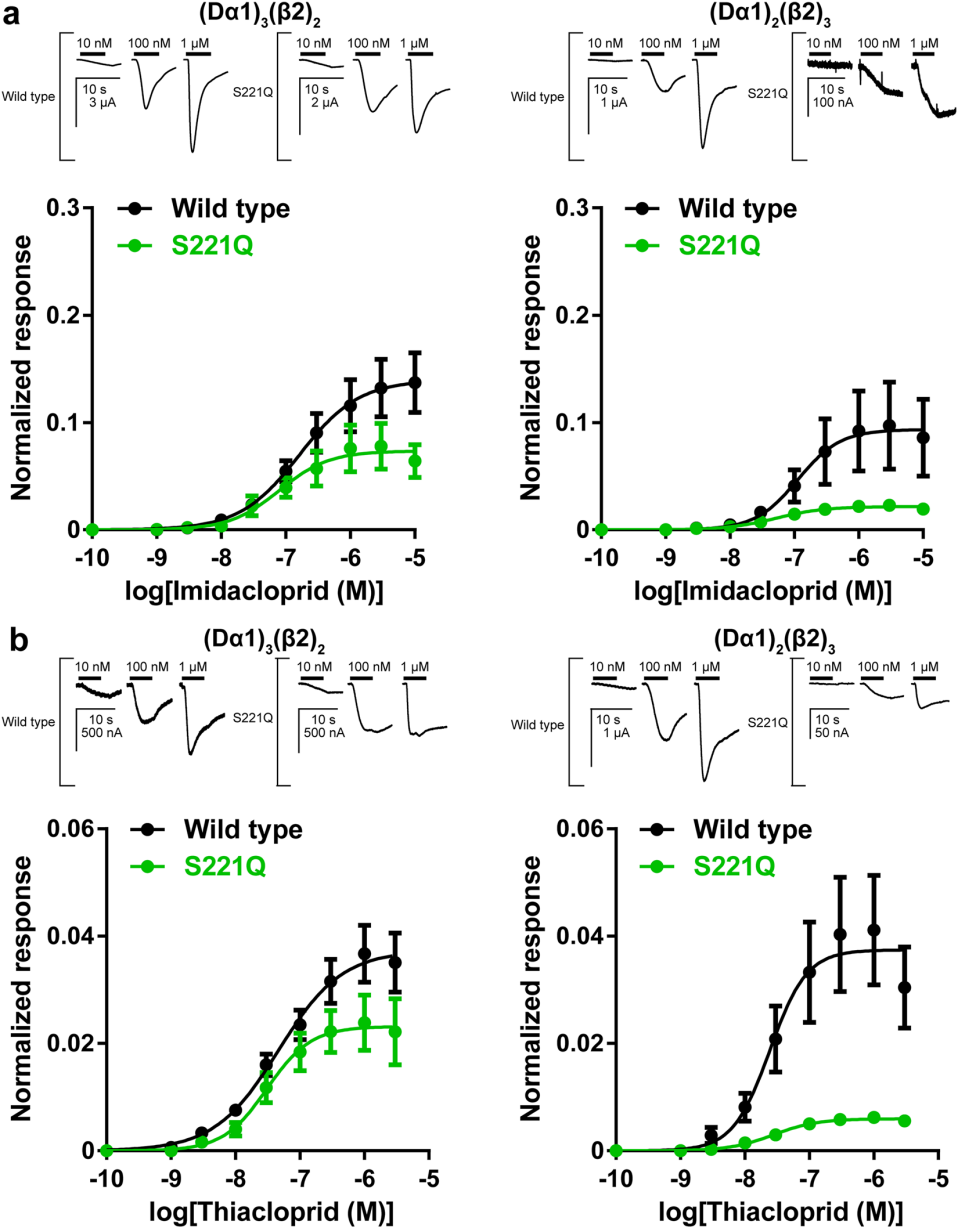
Agonist actions of imidacloprid and thiacloprid on wild type and S221Q mutant Dα1β2 nAChRs expressed in *Xenopus laevis* oocytes. Current responses to imidacloprid are shown above the concentration-response curves. Horizontal lines indicate bath applications of imidacloprid and thiacloprid. (**a**) Concentration-normalized response relationships of imidacloprid for the wild type and mutant (Dα1)_3_(β2)_2_ and (Dα1)_2_(β2)_3_ nAChRs. (**b**) Concentration-normalized response relationships of thiacloprid for the wild type and mutant (Dα1)_3_(β2)_2_ and (Dα1)_2_(β2)_3_ nAChRs. Each data plotted represents mean ± standard error of the mean (n = 5).

We previously hypothesized that the reduced affinity of the neonicotinoids by the S221E mutation in the Dα1/β2 nAChRs stems from electrostatic repulsion of the neonicotinoids containing a negative charge by the glutamate residue containing a negative charge^15^, as explained for the reduced agonist actions of imidacloprid on the Dα2/β2 nAChR by the P242E mutation in loop C^21^. To test this hypothesis, we investigated a mutation of Ser221 to glutamine having a similar size to glutamate but lacking a negative charge on the agonist activity of imidacloprid and thiacloprid on the hybrid nAChRs. The mutation was ineffective in changing the affinity but reduced the efficacy of imidacloprid in the (Dα1)_3_(β2)_2_ nAChR (*P* < 0.05, one-way ANOVA (Bonferroni-test)). Such effect on the agonist actions not only of imidacloprid, but also of thiacloprid, became more evident by increasing the β2 subunit composition ratio (Fig. 4a,b, Table 2, *P* < 0.05, one-way ANOVA (Bonferroni-test)). Hence, the reduced neonicotinoid affinity by the S221E mutation is mainly due to electrostatic repulsion by the glutamate residue, and that the reduced neonicotinoid efficacy in the (Dα1)_2_(β2)_3_ nAChR by Gln221 is due to its interference with neonicotinoid access to the Dα1/β2 interface by either steric interactions, or omission of the hydrogen bond or both.

### Models of Dα1/β2 nAChRs complexed with thiacloprid

**Figure 5:**
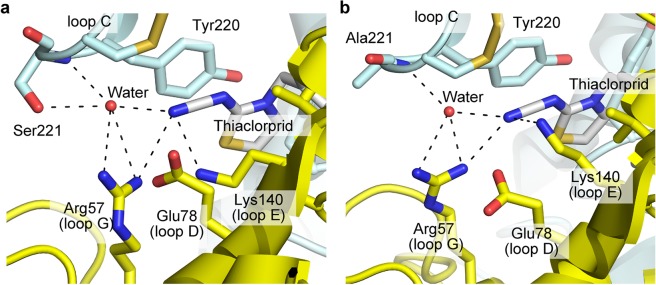
Models of Dα1/Dα1 subunit interface complexed with thiacloprid in (**a**) wild type and (**b**) S221A mutant (Dα1)_3_(β2)_2_ nAChRs. In the models, principal and complementary Dα1 subunits are illustrated as cartoon and colored pale cyan and yellow, respectively. Nitrogens, oxygen and sulfur atoms are colored blue, red and sand yellow, respectively, whereas carbons of thiacloprid are colored white. Electrostatic or hydrogen bond interactions are indicated by broken lines.

To further understand the role for Ser221 for the interaction with neonicotinoids, we modeled the Dα1/Dα1 subunit interface of the wild type and S221A mutant (Dα1)_3_(β2)_2_ nAChRs in complex with thiacloprid (Fig. 5), since the effects of the mutations on thiacloprid were greater than those on imidacloprid (Figs. 3 and 4). Thiazolidine ring of thiacloprid stacked with the aromatic ring of Tyr220, while the CN group interacted electrostatically with Arg57 in loop G and Lys140 in loop E and formed a hydrogen bond with the main chain NH group of Ser221 (Fig. 5). The hydroxy group of Ser221 formed hydrogen bond networks with the cyano group of thiacloprid as well as Arg57 and Lys140 (Fig. 5a). Thiacloprid can bind to the Dα1/Dα1 subunit interface in the S221A mutant because the basic residues in loop E and loop G support the binding (Fig. 5b), explaining the low impact of the mutation on the neonicotinoid action on the nAChRs with a higher composition ratio of Dα1 to β2. It is also suggested that when the mutations tested reduce the neonicotinoid sensitivity of the nAChRs with a higher composition of β2 to Dα1 because the β2 subunit does not possess any basic residue interacting with the nitro or cyano group of neonicotinoids and this would also be the case for imidacloprid^13^. The serine in loop C of the α1 subunit is encoded by AGC and AGT in *Anopheles gambiae* and *Locusta migratoria*, respectively, while being encoded by TCG and TCA in *Myzus persicae* and *Nilaparvata lugens*, respectively. We therefore predict that the serine to alanine mutation by a mutation of the first nucleotide T to G in *M. persicae* and *N. lugens* may occur and influence the neonicotinoid sensitivity of pests carrying the R81T mutation in loop D of the β1 subunit, and that the serine to glutamine mutation would occur less frequently than the serine to alanine mutation since two nucleotide mutations are needed. Further, we presume that a mutation of the serine to glycine may also occur by a mutation of the first nucleotide A to G, not only in the genome, but also in the mRNA^22^, and reduce the neonicotinoid sensitivity of *A. gambiae* and *L. migratoria*. As such, it is important to investigate whether these and related nucleotide mutations occur and affect the neonicotinoid sensitivity of pest insect species carrying the R81T mutation.

In conclusion, we have investigated the effects of S221A and S221Q mutations in loop C of the *Drosophila* Dα1 subunit on the agonist actions of ACh, imidacloprid and thiacloprid on the (Dα1)_3_(β2)_2_ and (Dα1)_2_(β2)_3_ nAChRs. Both mutations hardly lowered the affinity of the neonicotinoids. Hence, hydrogen bond capability of the serine residue has a minor contribution to the interactions with neonicotinoids in the insect/vertebrate hybrid nAChRs. However, the effects of these mutations on the efficacy of the neonicotinoids were evident in the (Dα1)_2_(β2)_3_ nAChR, pointing to a role for the serine in determining the neonicotinoid actions at the Dα1/β2 interface lacking the basic residues involved in the interactions with neonicotinoids. Although studies are needed to confirm this using nAChRs that are composed completely of insect nAChR subunits, the present results provided a new insight in the mechanism of selective actions of neonicotinoids and predicted resistance which may arise from the mutations tested in pest populations, notably with the R81T mutation.

## Data Availability

All data and material used in this study are available when requested.
